# Rhizosphere-Associated Bacteria of Saltgrass [*Distichlis spicata* (L.) Greene] Show Enhanced Ability to Tolerate Saline Environments and Stimulate Plant Growth

**DOI:** 10.3390/microorganisms13092046

**Published:** 2025-09-02

**Authors:** Ángel Mena-García, Alejandro Alarcón, Fernando C. Gómez-Merino, María G. Peralta-Sánchez, Libia I. Trejo-Téllez

**Affiliations:** Colegio de Postgraduados Campus Montecillo, Carretera México-Texcoco km 36.5, Montecillo, Texcoco C. P. 56264, State of Mexico, Mexico; angelmegar@hotmail.com (Á.M.-G.); alexala@colpos.mx (A.A.); fernandg@colpos.mx (F.C.G.-M.); mgperalta@colpos.mx (M.G.P.-S.)

**Keywords:** microbial biostimulant, salinity, plant growth-promoting bacteria, biostimulation

## Abstract

The use of plant growth-promoting bacteria (PGPB) tolerant to abiotic stress factors can enhance plant performance when applied under both optimal and stress conditions in crops. In this study, bacterial strains associated with the rhizosphere of the halophyte *Distichlis spicata* were isolated and characterized for their ability to produce siderophores, solubilize phosphate, synthesize indole-3-acetic acid (IAA) and exopolysaccharides (EPS), and tolerate salinity. IAA production and antioxidant capacity were further assessed under saline stress. As expected, salinity negatively impacted bacterial growth, IAA biosynthesis, and antioxidant activity—even in strains from a salt-tolerant plant. Nevertheless, all strains except RD2 maintained growth and IAA production in LB broth supplemented with up to 1 M NaCl. Five halotolerant strains (RD2, RD4, RD17, RD26, and RD27) were selected for greenhouse inoculation assays in tomato (*Solanum lycopersicum*) seedlings. Inoculation with RD26 significantly enhanced seedling performance, promoting tomato growth, increasing leaf area by 22%, stem diameter by 17%, shoot dry biomass by 30%, and root biomass by 27% as compared to the uninoculated control. RD27 and RD4 also improved shoot biomass by 25 and 23%, respectively. Based on 16S rRNA gene sequencing, RD26 was identified as *Pseudomonas* sp. and RD27 as *Zhihengliuella halotolerans*. These findings demonstrate that salt stress impairs plant growth-promoting traits in rhizospheric bacteria, yet selected strains such as RD26 and RD27 can significantly promote plant growth. Their use as bioinoculants represents a promising strategy for improving crop performance in saline environments.

## 1. Introduction

The agricultural sector has a growing need to develop more sustainable and soil-friendly production systems and to explore new environments in search of tools that aid plant growth. Plant growth-promoting bacteria (PGPB) are a group of beneficial bacteria that act as biofertilizers or biostimulants and associate with plants in the rhizosphere, on the root surface, or endophytically within their tissues [1].

These bacteria promote plant growth through diverse mechanisms, either directly, such as by nitrogen fixation [2,3], phosphate solubilization [4,5], or the production of siderophores and phytohormones [6,7,8], or indirectly, by suppressing plant pathogens via antimicrobial compound synthesis or by competing for space and nutrients in the rhizosphere [9,10]. Some halotolerant PGPB may also alleviate salt-induced oxidative stress in plants through the production of antioxidant compounds, such as phenolic metabolites or enzymes that scavenge reactive oxygen species (ROS) [11,12]. These traits have been widely reported in strains associated with both conventional crop plants and non-cultivated halophytes.

The crop rhizosphere is the most studied area for obtaining bacteria with potential traits associated with plant growth promotion; however, in some cases, when these bacteria are subjected to environmental stresses such as salinity, their beneficial capacities can be hampered. For example, *Azospirillum brasilense*, *Pseudomonas fluorescens* [4], and *Bacillus pumilus* JPVS11 [6] gradually reduced the synthesis of indoleacetic acid (IAA) in the culture medium as the NaCl level increased. Nonetheless, *Alcaligenes* sp. AF7 exposed to salt stress increased its antioxidant capacity [13]. Exploration of the rhizosphere of halophytic plants has shown it to be a habitat for bacterial strains capable of expressing their growth-promoting traits in vitro under salt stress [2]. Those bacterial strains are able to associate with non-halophytic plants or crops that are not tolerant to salinity to promote growth [9]. In some cases, the isolation of strains from the rhizosphere of halophytes can also be affected by salinity and reduce the expression of biochemical traits such as IAA production or decrease their effectiveness in improving crop growth under non-stress conditions, since NaCl can stimulate the production of metabolites through which bacteria promote plant growth [2].

The identification of bacteria for their application as potential inoculants through traits that can promote plant growth is performed under in vitro conditions and follows a sequence of isolation and cultivation in selective media, with the aim of confirming the production of metabolites or the presence of specific functional groups that may have a plant growth-promoting effect [14].

Saltgrass [*Distichlis spicata* (L.) Greene] is a herbaceous species from the Poaceae family that thrives in environments with extreme salinity, where most plant species cannot survive. In addition, it exhibits remarkable drought tolerance, being able to endure prolonged periods of water scarcity. This adaptive capacity is achieved through the regulation of cellular concentrations of harmful salts, osmotic regulation, maintenance of cell turgor, germination in times of low water stress, and the association with arbuscular mycorrhizal fungi (AMF) that help regulate metabolism and explore soil areas with higher water and nutrient content [15]. In addition, this grass is associated with beneficial bacteria that play a fundamental role in plant nutrition and in the regulation of metabolic pathways that control abiotic stress, such as the activity of the enzyme 1-aminocyclopropane-1-carboxylic acid deaminase (ACC deaminase) [9].

The identification of beneficial bacteria associated with *D. spicata* has been previously conducted through in vitro inoculation assays using *Arabidopsis thaliana*, from which several rhizospheric and endophytic strains were isolated and evaluated [9]. Some of these strains, such as *Bacillus* sp. and *Pseudomonas lini*, showed growth-promoting effects and enhanced salt tolerance in *Arabidopsis*, cucumber (*Cucumis sativus*), and watermelon (*Citrullus lanatus*), highlighting their potential across diverse plant species. However, little is known about how salinity affects the maintenance of key PGPB traits in these strains, such as IAA synthesis, siderophore production, antioxidant activity, and exopolysaccharide (EPS) secretion. These beneficial traits are typically characterized under laboratory conditions, but their expression may vary under abiotic stress, such as salinity. In this study, we focus on how specific PGPB traits—especially IAA production, siderophore synthesis, antioxidant activity, and EPS production—are maintained under salt stress by rhizobacteria isolated from halophytic environments.

We hypothesized that certain rhizobacteria associated with *D. spicata* retain their plant growth-promoting traits under salt stress and can enhance seedling development in tomato (*Solanum lycopersicum*) when used as inoculants. Therefore, the objective of this research was to isolate and characterize rhizospheric bacteria from *D. spicata* that retain plant growth-promoting properties in vitro under salt stress and to evaluate their effectiveness in promoting growth in tomato (*Solanum lycopersicum*) seedlings under controlled greenhouse conditions.

## 2. Materials and Methods

### 2.1. Sampling Area

**Figure 1:**
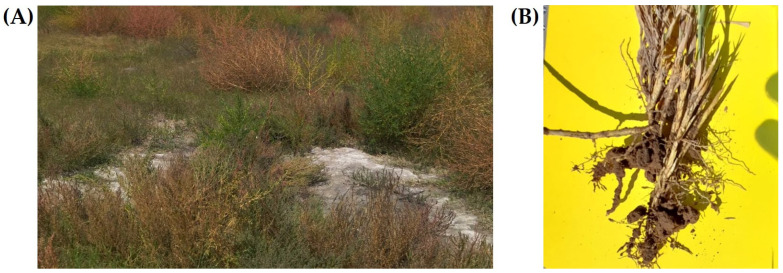
Sampling area in Montecillo, Texcoco, State of Mexico, Mexico, for bacteria isolation (19°27′56.3″ N, 98°55′2.4″ W, at 2240 m elevation). (**A**) General view of the sampling site, (**B**) soil adhered to the roots of saltgrass (*Distichlis spicata*) used for bacterial isolation.

Sampling was conducted in the former Lake Texcoco area, specifically in Montecillo, Texcoco, in the State of Mexico (19°27′56.3″ N, 98°55′2.4″ W). A plot dominated by saltgrass (*Distichlis spicata*) and romerito (*Suaeda* sp.), both considered indicator species of saline environments [16], was selected for this study (Figure 1A).

Two independent samplings were performed: one for bacterial isolation and another for the analysis of soil chemical properties. For bacterial isolation, a composite soil sample was obtained by mixing five subsamples collected from the rhizosphere of *D. spicata*. Whole plants were carefully removed with their roots intact, and excess soil loosely attached to the root system was shaken off (Figure 1B). Then, the rhizosphere soil—defined as the soil tightly adhering to the roots after shaking—was detached by gently brushing or scraping it from the root surface using sterile spatulas. This material was collected into airtight bags previously sterilized with 70% alcohol, thoroughly mixed, and stored at 4 °C until further processing.

Soil sampling was performed using a shovel disinfected with 70% alcohol at a depth of 20 cm near the plant root exploration zone. A composite sample was obtained by combining five subsamples. The collected soil was thoroughly mixed and spread over a plastic sheet, divided into four equal sections, and the two opposite sections were discarded. The remaining soil was mixed again, and this quartering procedure was repeated until approximately 1.5 kg of soil was obtained.

### 2.2. Soil Analysis

The soil samples were air-dried at room temperature and sieved through a 10-mesh (2 mm) sieve prior to analysis. Chemical properties were determined following the official Mexican standard NOM-021-RECNAT-2000 [17], which outlines standardized methods for soil fertility assessment and classification. The evaluated parameters included organic matter content (Walkley–Black method), soil pH (measured in a 1:2 soil-to-water suspension), and electrical conductivity (EC, determined from the saturated paste extract). Cation exchange capacity (CEC) and exchangeable bases (Ca^2+^, Mg^2+^, Na^+^, and K^+^) were determined using sodium acetate extraction, which is suitable for alkaline soils. Soil salinity was also evaluated using the saturated paste extract method. All analyses were performed in duplicate under controlled laboratory conditions.

### 2.3. Bacterial Isolation

The dilution and spread plate method was used for bacterial isolation [18]. A 100 g sample of rhizosphere soil was moistened with sterile distilled water and left to stand for 24 h to reactivate microbial cells and enhance their release from soil particles, thereby improving bacterial recovery. Subsequently, a 10 g sample of moist soil was mixed with 90 mL of sterile distilled water and kept under constant stirring at 180 rpm for 30 min in a shaking incubator (Maxq 4000, Thermo Scientific; Holtsville, NY, USA). After the incubation period, serial dilutions were made. Then 100 µL aliquots of the 10^−3^, 10^−4^, and 10^−5^ dilutions were plated and spread in triplicate on nutrient agar (NA) plates (BD Bioxon; Cuautilán Izcalli, State of Mexico, Mexico) supplemented with 0, 0.25, 0.5, 0.75, and 1 M NaCl (Meyer; Mexico City, Mexico). The plates were incubated for 72 h at 28 °C in an incubator (Model G|11, Sheldon Manufacturing; Cornelius, OR, USA), and colonies with different morphologies were purified in NA. Subsequently, the bacteria were subjected to a salinity tolerance test in NA supplemented with 0, 0.25, 0.50, 0.75, 1, 2, and 3 M NaCl.

In some of the biochemical tests, a strain of *Arthrobacter pokkalii* was used as a reference, a bacterium isolated from the rhizosphere of tomato (*Solanum lycopersicum* L.) identified as JLB4, which solubilizes tricalcium phosphate, produces IAA, and improves the growth of this crop at the seedling stage [19].

### 2.4. Qualitative In Vitro Tests to Select Bacteria with Plant Growth-Promoting Traits

Siderophore production. The chrome azural S (CAS) culture medium described by Louden et al. [20] was used. The methodology for siderophore determination can be performed from liquid culture; however, hexadecyltrimethylammonium bromide (HDTMA) can interfere with the growth of bacteria inoculated at low concentrations; therefore, an aliquot of the bacterial colony was directly inoculated into the CAS medium. From a young culture grown in NA, an aliquot was taken and directly seeded in CAS medium in a circular shape. The dishes were incubated for 5 d at 28 °C. The formation of a yellow or orange halo around the bacterial growth was considered a positive reaction to siderophore production.

Phosphate solubilization. Phosphate solubilization production capacity was carried out on solid culture medium Pikovskaya [21], the composition of which in g L^−1^ is as follows: 10 glucose (Sigma-Aldrich; St. Louis, MO, USA), 5 Ca_3_(PO_4_)_2_ (Sigma-Aldrich; Schnelldorf, Germany), 0.5 (NH_4_)_2_SO_4_ (Merck; Darmstadt, Germany), 0.2 NaCl, 0.1 MgSO_4_ 7H_2_O (Meyer; Mexico City, Mexico), 0.2 KCl (J. T. Baker; Radnor, PA, USA), 0.5 yeast extract (Merck; Darmstadt, Germany), traces of MnSO_4_ H_2_O (J. T. Baker; Mexico City, Mexico), traces of FeSO_4_ 7H_2_O (J. T. Baker; Mexico), 15 agar (BD Bioxon; Cuautitlán Izcalli, State of Mexico, Mexico), and pH 7.0. The strains were streaked onto the medium, and the formation of a halo around the bacterial growth was evaluated, which indicated the secretion of organic acids.

IAA synthesis (total indoles). The methodology described by Gordon and Weber [22] was used with some modifications. One inoculation loopful of each of the bacteria was grown in triplicate in 20 mL of Luria–Bertani (LB) broth supplemented with 1 g tryptophan L^−1^ (Meyer; Mexico City, Mexico) contained in 50 mL borosilicate flasks. The LB broth contains in g L^−1^: 10 of tryptone (Merck; Darmstadt, Germany), 5 of NaCl, and 5 of yeast extract. The flasks were grown for 48 h at 160 rpm at 28 °C. After the incubation period, the bacterial cell biomass was separated from the supernatant by centrifugation at 5204× *g* for 10 min in a centrifuge (Sigma Laborzentrifugen 2-16P; Harz, Germany). One mL of bacterial supernatant was mixed with 2 mL of Salkowski reagent [2% 0.5 M FeCl_3_ (Fermont; Monterrey, NL, Mexico) in 35% HClO_4_ (J. T. Baker; Radnor, PA, USA)]. Samples with pink coloration were classified as positive to produce indole auxins.

Exopolysaccharide (EPS) production. EPS production was assessed using LB Congo Red (LB-CR) medium supplemented with 5% sucrose or glucose (Sigma-Aldrich; St. Louis, MO, USA) as the carbon source. A loopful of 48 h-old bacterial cultures grown in NA was plated in triplicate onto plates containing LB-CR with glucose or sucrose and incubated for 5 d at 28 °C. Strains that reacted with the CR were recorded as EPS positive, as evidenced by color change in the medium and colonies from red to black [23].

### 2.5. Quantitative Tests Under Salt Stress

IAA quantification. The strains were plated in LB broth and incubated at 28 °C with constant shaking at 180 rpm for 24 h. After the incubation period, a 100 µL sample of culture with an absorbance of 0.4 (OD 600 nm) was grown in 20 mL of LB broth supplied with 1 g tryptophan L^−1^ (Meyer; Mexico City, Mexico) contained in 50 mL borosilicate glass bottles. The strains were incubated at 28 °C at 180 rpm for 48 h. A 1.5 mL sample was taken and placed in 2 mL microcentrifuge tubes (Axygen^®^; St. Louis, MO, USA) and centrifuged at 4600× *g* for 10 min in a centrifuge (Heraeus FrescoTM 17, Thermo Scientific; Hamburg, Germany). A 100 µL sample of supernatant was mixed with 200 µL of Salkowski reagent in microplate wells for spectrophotometry. The samples were allowed to react for 30 min in the dark with constant stirring and were read at 530 nm in a spectrophotometer (Synergy 2 Microplate Reader, BioTek; Winooski, VT, USA). A standard curve prepared in LB broth from IAA (Sigma-Aldrich, Buchs, Switzerland) and processed under the same experimental conditions was used to determine the auxin concentration. The values are reported in µg mL^−1^.

Antioxidant activity (DPPH scavenging). The 2,2-diphenyl-1-picrylhydrazyl (DPPH) radical scavenging activity was determined following the methodology described by Xing et al. [24], with minor modifications. Bacterial cultures were grown in nutrient broth (BD Bioxon; Cuautitlán Izcalli, State of Mexico, Mexico) for 48 h at 28 °C with constant shaking at 160 rpm. The culture was adjusted to an optical density of 0.4 at 600 nm (OD_600_), and a 100 µL aliquot was used to inoculate 50 mL borosilicate glass flasks containing 20 mL of nutrient broth supplemented with 0, 0.25, 0.5, 0.75, and 1 M NaCl (Meyer; Mexico City, Mexico). These flasks were incubated under the same salinity conditions for 48 h at 28 °C and 180 rpm.

After the incubation period, the cultures were centrifuged at 4600× *g* for 10 min at 4 °C in a microcentrifuge (Heraeus Fresco^TM^ 17, Thermo Scientific; Hamburg, Germany) to collect the supernatant. The supernatant was then filtered through a 0.22 µM membrane and used to measure the DPPH scavenging activity. For the assay, 100 µL of the filtrate was mixed with 100 µL of 0.2 mM DPPH solution prepared in methanol (Sigma-Aldrich; Seelze, Germany) in 96-well microplate wells. The mixture was incubated in the dark with constant shaking for 30 min. Absorbance was measured at 517 nm using a microplate spectrophotometer. Nutrient broth mixed with DPPH solution served as a control. Antioxidant activity was determined based on the following formula described by Fatima et al. [13]:

DPPH scavenging activity (% removal) = [(A control − A sample)/A control)] × 100

A control = absorbance of the control sample

A sample = absorbance of the filtrate sample

### 2.6. Greenhouse Assay and Molecular Identification

An experiment with a completely randomized design was established under greenhouse conditions to evaluate the effect of bacterial inoculation on tomato seedlings. Seven treatments were included: five bacterial isolates (RD2, RD4, RD17, RD26, RD27), one uninoculated control, and a reference strain (*Arthrobacter pokkali*, JLB4), previously isolated from the tomato rhizosphere [19]. Each treatment had four replicates, and each experimental unit consisted of six tomato seedlings grown in a sterilized peat-vermiculite (80:20, *v*/*v*) substrate.

Tomato seeds were surface-sterilized by immersion in 1.5% sodium hypochlorite for 3 min and rinsed three times with sterile distilled water. Tomato seeds were germinated under sterile conditions, and after the development of the second true leaf, seedlings were inoculated with 2 mL of bacterial suspension adjusted to an optical density of OD_600_ = 0.5.

Bacterial cultures were prepared by growing each isolate in NA for 48 h at 28 °C, followed by incubation in LB broth for 3 days at 28 °C with constant shaking. Cells were harvested by centrifugation and resuspended in sterile distilled water prior to inoculation.

Following inoculation, plants were irrigated alternately with sterile distilled water and 25% Steiner nutrient solution [25] for 30 days. Agronomic traits were evaluated at the vegetative stage (36 days post-inoculation), including leaf area, stem diameter, and dry biomass of shoots and roots.

Molecular identification of the two most effective strains (RD26 and RD27) was performed by sequencing the 16S rRNA gene. Genomic DNA was extracted following a modified phenol-chloroform protocol. PCR amplification was carried out using universal primers, and products were purified and sequenced (Macrogen; Rockville, MD, USA). The resulting sequences were analyzed using the BLAST+ version 2.15 tool [26].

### 2.7. Statistical Analysis

Statistical analyses were performed using R software version 4.1.1 [27]. For most in vitro bacterial assays, results were based on six replicates (n = 6) per treatment. However, for bacterial growth analyses, three replicates (n = 3) were used. For plant-based experiments, four biological replicates (n = 4) were used per treatment.

The data for each variable were subjected to Shapiro–Wilk tests to evaluate the assumption of normality and Levene tests to assess homogeneity of variances (α = 0.05). Variables that met the assumptions of normality and homoscedasticity were analyzed using one-way ANOVA, followed by Tukey’s post hoc test (*p* ≤ 0.05) for multiple comparisons.

For data that violated ANOVA assumptions, Box–Cox or square root transformations were applied, and the analyses were repeated. When transformations failed to normalize the data, non-parametric alternatives were used: variables were ranked and analyzed using Kruskal–Wallis tests, with pairwise comparisons interpreted based on the original means.

Correlation analyses were performed only for IAA production and bacterial density under salt stress. In this case, Pearson’s correlation was applied to normally distributed data.

## 3. Results and Discussion

### 3.1. Chemical Properties of the Soil

The bacterial isolates were obtained from a soil with an electrical conductivity in the saturated paste extract of 30.34 dS m^−1^ and with an exchangeable sodium percentage (ESP) of 58.8, which classifies it as a sodic saline soil; also, the pH value of 10.8 indicates the predominance of exchangeable sodium [28]. The results of the chemical analysis of soil fertility are shown in Table 1 and Table 2.

### 3.2. Bacterial Isolations

A total of 29 strains were isolated from the rhizosphere of saltgrass. These strains were designated with the RD code and a consecutive number, indicating that they were isolated from the rhizosphere of *Distichlis spicata*.

The salinity tolerance assay in NA supplemented with 0, 0.250, 0.500, 0.750, and 1 M NaCl indicated that all bacteria grew in the presence of up to 0.750 M NaCl, while in the presence of 3 M NaCl, none of the bacteria grew (Table 3).

### 3.3. Evaluation of Plant Growth-Promoting Traits

Of the 30 isolated strains, 24 were selected for their degree of salinity tolerance in the qualitative NA assay. Each of the 24 selected strains was identified as capable of producing EPS in the presence of sucrose and glucose as carbon sources, as well as synthesizing IAA, solubilizing phosphate, and synthesizing siderophores. Of these strains, four synthesized EPS in the presence of sucrose and four in the presence of glucose as a carbon source; five produced IAA and siderophores, but none solubilized phosphate (Table 4).

### 3.4. Siderophore Production and Phosphate Solubilization

Phytosiderophore synthesis is part of the so-called Strategy II of species in the Poaceae family for Fe acquisition, especially when they grow under conditions that limit said element. In non-grass plants, Fe uptake can be enhanced by the application of synthetic siderophores or directly and indirectly by biological siderophores [29].

In the soil, in the rhizosphere, and in culture tissues in an endophytic manner, there are bacteria that synthesize siderophores naturally and excrete them to the cell exterior to chelate metals [30]. In bacteria, as in plants, the production of these organic molecules intensifies in response to stress due to Fe deficiency or in the presence of unavailable trivalent forms [31]. The isolation and identification of siderophore-producing bacteria (SPB) in CAS medium at 5 d indicated that strains RD2, RD4, RD11, RD17, RD26, RD27, and R29 changed the color of the medium and formed a yellow-orange halo around the colony, indicating the production of siderophores [31] (Figure S1, Supplementary Materials). Strain RD26 showed a greater capacity to produce siderophores. There are several factors that can affect siderophore production under controlled conditions, including the HDTMA in the culture medium, which is toxic to some bacterial growth; the source of C used; and the pH and concentration of trivalent Fe that will be used as a substrate by the bacterial siderophores [31]. The greater production of yellow halo by strain RD26 compared to the rest of the strains may be a result of the high capacity to synthesize them and respond to Fe-limiting conditions or due to its adaptation to the pH of the medium, to the use of glucose as a carbon source, or to its tolerance to HDTMA.

Siderophore-producing bacteria are also associated with the rhizosphere of halophytic plants, as is the case with the rhizosphere of *D. spicata*, from which two bacterial strains were identified, *Bacillus* sp. LBEndo1 and *Pseudomonas lini* KBEcto4, with the ability to synthesize siderophores in CAS culture medium [9]. Similarly, strains belonging to the genus *Bacillus* were isolated from the rhizosphere of sea aster (*Tripolium pannonicum*, formerly *Aster tripolium*) with the ability to chelate Fe [30].

In environments with high salinity and alkaline pH, barley (*Hordeum vulgare* L.) plants increase the synthesis of siderophores to ensure their supply of Zn. This increase in synthesis is caused by the reduction in the capacity of siderophores to efficiently bind to divalent or trivalent metals, so higher concentrations of these compounds are required to compensate for the chelation potential normally expressed in situations of normal growth without salinity and with Fe deficiency. Therefore, it is necessary to supply the crop requirements, even when the plants do not experience a nutritional shortage of Fe in the growth medium [32].

Obtaining strains from the rhizosphere of saline environments capable of producing siderophores for application in crops subjected to salt stress could ensure greater guarantees of success in improving not only Fe nutrition for plants but also other micronutrients that have affinity with bacterial siderophores. For example, in *Bacillus aryabhattai* MS3, the *entD* gene was expressed even under salt stress at 200 mM NaCl in a situation of Fe limitation but was completely inhibited when the Fe concentration in the medium was increased, independently of the NaCl concentration [7]. Furthermore, these data suggest that bacteria can provide Fe to plants even in environments with high ionic and osmotic pressure in the growth substrate.

In the present study, the bacterial strains did not show the ability to solubilize phosphate after 7 and 14 d (Figure S2, Supplementary Materials). Phosphate-solubilizing bacteria do so mainly through the synthesis of exopolysaccharides or by excreting siderophores. Siderophores release phosphate by chelating Fe^3+^, Ca^2+^, and Al^3+^ cations with which phosphate forms poorly soluble complexes that plants cannot directly absorb from the soil [5]. The bacteria isolated in this work and those that produced siderophores did not solubilize phosphate. It has been previously reported that not all siderophore-synthesizing bacteria solubilize phosphate [33]. This may be because several factors affect siderophore synthesis, such as the N source, Fe availability, and the use of organic acids as a carbon source [8]. Furthermore, siderophores are characterized as having a higher affinity for Fe^3+^ than Fe^2+^ and Ca^2+^, so the results obtained in this work may be associated with the fact that the type of siderophore produced by the isolated bacteria does not have an affinity for Ca^2+^, the accompanying cation of the phosphate source used to determine the phosphate solubilization capacity.

### 3.5. Exopolysaccharide (EPS) Production

Bacterial EPS production is one of the traits evaluated in bacteria living in saline environments. The use of EPS-producing bacteria as inoculum or their purified EPS has similar effects on promoting plant growth. Evaluations in LB-RC solid culture medium supplemented with two carbon sources showed that EPS production capacity varied depending on the carbon source used. Of the 24 strains tested, only RD10, RD11, RD28, and RD29 produced EPS at 5 d in the presence of sucrose as a carbon source. When the medium was supplemented with glucose, the strains decreased EPS synthesis, although with this carbon source, strains RD4, RD10, RD14, and RD29 excelled in EPS production (Figures S3 and S4, Supplementary Materials).

EPS produced by rhizospheric bacteria are used to colonize and adhere to roots and form mutualistic associations with plants [34,35] or to protect themselves and survive abiotic phenomena such as drought and salinity [34]. In the soil solution, EPS-producing bacteria remove Na^+^ from the liquid phase and develop an organic membrane that surrounds the root zones and acts as a physical barrier preventing the entry of Na^+^. The hydroxyl functional groups of carbohydrates and carboxyl groups of proteins that make up the EPS function as organic sorbents of Na^+^, which, by binding it, reduce its availability in the soil and ionic toxicity in roots [10,36]. Moreover, sodium adsorption to EPS surfaces is favored under neutral or basic pH, where functional groups are in their deprotonated form, enhancing cation-binding capacity via electrostatic attraction [37].

In *Yersinia enterocolitica*, the biosynthesis of EPS and biofilm formation are regulated by signaling pathways responsive to environmental cues such as changes in osmolarity. Under low-salt conditions, reduced synthesis of osmoregulated periplasmic glucans (OPGs) triggers activation of the Regulator of Capsule Synthesis (Rcs) phosphorelay system, which represses motility-related genes (*flhDC*) and biofilm-associated genes (*hmsT*, *hmsHFRS*), while also decreasing intracellular cyclic-di-GMP levels. These findings suggest that the Rcs system acts as an osmotic stress sensor that modulates bacterial physiological responses to environmental fluctuations [38]. In other bacterial species, alternative sigma factors can also dynamically reprogram transcription in response to environmental prompts, including osmotic stress [39].

Under saline conditions, EPS production enhances bacterial survival by facilitating biofilm formation and increasing water retention in the rhizosphere, thereby maintaining microenvironments with lower ionic strength [40]. This physiological adaptation not only supports bacterial fitness but also contributes to plant tolerance to salt stress, as EPS can bind sodium ions, reducing their availability for root uptake. Furthermore, halotolerant rhizobacteria capable of forming stable biofilms have been shown to alleviate salinity stress in tomato plants, improve antioxidant defenses, and regulate ionic balance, reinforcing the role of EPS in rhizosphere stabilization under saline conditions [41].

EPS-producing plant growth-promoting rhizobacteria (PGPR) strains have been shown to decrease Na^+^ uptake in wheat (*Triticum aestivum* L.) by binding sodium ions in the rhizosphere and enhancing soil aggregation around roots, ultimately improving plant biomass under salinity stress [42].

### 3.6. IAA Production and Bacterial Growth

**Figure 2:**
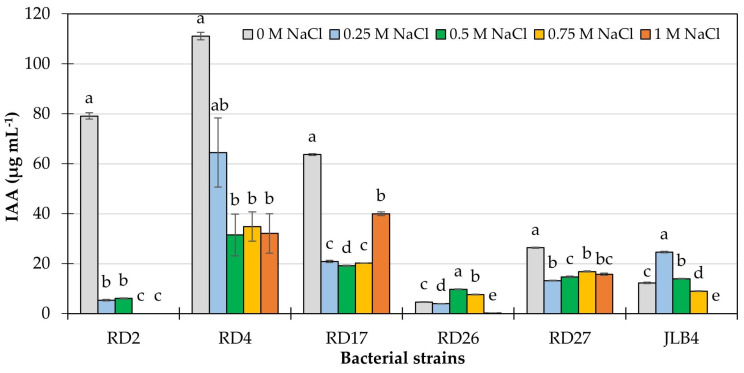
Indoleacetic acid (IAA) production by five bacterial strains isolated from the rhizosphere of saltgrass (*Distichlis spicata*), compared to the reference strain JLB4 (*Arthrobacter pokkalii*), under salt stress conditions. Mean ± SE with different letters indicates statistically significant differences between treatments (Tukey, *p* ≤ 0.05), *n* = 6.

IAA synthesis occurs in both plants and bacteria through pathways dependent and independent of L-tryptophan. Soil bacteria capable of synthesizing IAA find their habitat in the rhizosphere or within the tissues of halophytes [43]. In vitro detection of IAA is one of the most common procedures used in the study of beneficial microorganisms to determine their potential use as an alternative in sustainable crop management [11,12,44,45]. The capacity for IAA synthesis and secretion in saline media varies between bacterial species, and the presence of NaCl in the growth medium can reduce, increase, or maintain it unchanged [3]. In the present study, IAA production was examined in five strains isolated from the rhizosphere of saltgrass under both normal and NaCl-induced salt stress conditions. All five bacteria were previously identified as positive for IAA synthesis in a qualitative colorimetric assay using tryptophan as a precursor. All isolated strains showed significant levels of IAA in the culture broth supernatant under normal physiological growth conditions, meaning without induced salt stress (Figure 2), where strain RD4 produced the highest concentration of IAA (111.09 µg mL^−1^), compared to the rest of the isolated strains and the reference strain JLB4, which exhibited lower values. On the other hand, strain RD26 showed the lowest production, with 4.60 µg IAA mL^−1^. Conversely, it has been previously reported that two bacterial species isolated from the rhizosphere of *D*. *spicata* that thrive in northern Mexico showed lower concentrations of IAA produced under normal growth conditions in the presence of L-tryptophan; the *Bacillus* sp. LBEndo1 and *Pseudomonas lini* KBEcto4 strains synthesized 23.44 and 36.6 µg IAA mL^−1^, respectively [9].

These differences in IAA production may be attributed to how bacterial metabolic pathways respond to osmotic and ionic stress caused by salinity. Under high NaCl concentrations, the energetic burden of maintaining osmotic homeostasis may divert metabolic resources away from secondary metabolite biosynthesis, including IAA. Such physiological constraints under salt stress have been reported to alter the metabolic activity of halotolerant bacteria, affecting their ability to promote plant growth through IAA synthesis and other mechanisms. These responses are strain-specific and reflect the differential halotolerance observed among isolates [46,47]. Our observations suggest that these responses are strain-specific and reflect differences in halotolerance among the tested isolates.

The supply of high concentrations of NaCl in the culture medium caused a marked alteration in IAA synthesis in all strains. In bacteria RD2, RD4, RD17, and RD27, IAA synthesis decreased in the presence of salinity; however, strains RD4, RD17, and RD27 were able to synthesize IAA at all NaCl concentrations tested. In the case of strain RD2, the supply of 0.25 M NaCl reduced IAA synthesis by 93%, with total inhibition observed starting at 0.75 M NaCl. The application of 1 M NaCl inhibited complete IAA production in JLB4 and RD26; however, in JLB4, the highest IAA concentration was obtained with the addition of 0.25 M NaCl with 24.59 µg mL^−1^ (Figure 2). In some bacterial species, the application of NaCl can even stimulate IAA production. For example, *Bacillus mycoides* PM35 exposed to 900 mM NaCl increased IAA synthesis by 20% compared to the control without NaCl [12]. In contrast, in *Azospirillum brasilense*, *P. fluorescens* [4], and *Bacillus pumilus* JPVS11, IAA concentration gradually decreased as NaCl concentration increased [6].

**Figure 3:**
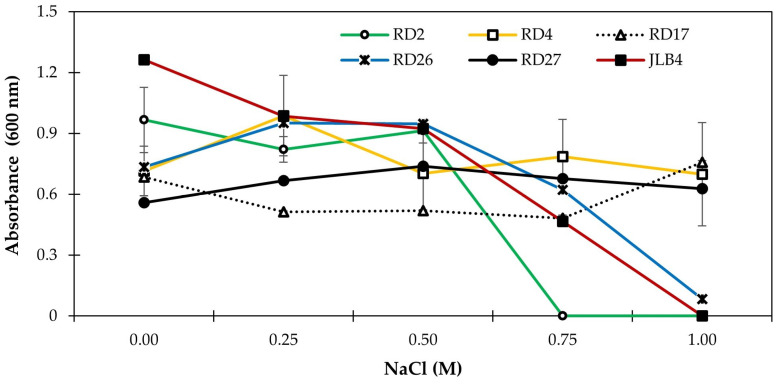
Salinity tolerance of five bacterial strains isolated from the rhizosphere of saltgrass (*Distichlis spicata*) and the reference strain JLB4 (*Arthrobacter pokkalii*) in LB broth supplemented with tryptophan. Data represent mean values ± SE, n = 3.

To determine whether IAA production was associated with the adaptive capacity of bacteria or with interference in the metabolism of the IAA synthesis pathway by the ionic and osmotic effects of NaCl, bacterial growth was measured by the turbidimetric method in a spectrophotometer in the culture medium from which the bacterial filtrates were obtained to measure IAA (Figure 3). A similar response in bacterial growth was found in IAA production under salt stress. Strains RD4, RD17, RD26, and RD27 showed continuous growth even at the highest NaCl concentration, whereas strain RD2 maintained growth up to 0.5 M NaCl and was completely inhibited at the highest concentrations. In RD26, the highest growth was obtained at 0.25 and 0.5 M NaCl, whereas in strain JLB4, growth was reduced as the level of NaCl in the culture medium increased and was completely inhibited in the presence of 1 M NaCl (Figure 3).

Halotolerant and halophilic bacteria regulate their metabolism and manage to survive high osmotic pressures through the ability to synthesize organic compounds that act as osmolytes that help maintain cell turgor and acquire water under these conditions [48]. Proline, glycine betaine, ectoine, and trehalose are some of the metabolites produced by bacteria that inhabit saline soils or that are associated with halophytic plants [1,6,49,50]. These osmolytes are important for bacteria because they help them survive salt stress and also promote plant growth. Interestingly, the addition of 0.25 and 0.5 M NaCl stimulated the growth of strain RD26 by more than 30% and reduced it from 0.75 M onwards. In the presence of moderate concentrations, NaCl can stimulate cellular multiplication of PGPB. In *Azospirillum,* the addition of 0.1 M NaCl had a beneficial effect on cell multiplication [51], whereas in the non-pathogenic strain of *Enterobacter cloacae*, the addition of NaCl can promote cell growth and reduce it when it exceeds the tolerance threshold [45]. This indicates that bacteria can be induced at low NaCl concentrations and that growth is reduced when their salinity tolerance threshold is exceeded.

The Spearman correlation analysis showed significance (*p* ≤ 0.05) between IAA production and growth of the RD2 strain under NaCl supplementation. This finding suggests that the reduction in IAA in the presence of NaCl in RD2 is associated with the effect of salinity on bacterial density, as previously reported in *Azotobacter chroococcum* and *Bacillus subtilis*, where the amount of IAA produced from tryptophan increased as a function of the increase in the number of cells in the medium and decreased after the stationary phase, when the bacteria enter the death phase. Likewise, IAA production in some bacterial species can occur from 12 h and reach a maximum up to 96 h and subsequently reduce as dead cells increase [52].

On the other hand, there was no correlation between IAA production and the growth of strains RD4, RD17, RD26, RD27, and JLB4 in the medium under salt stress. This lack of correlation suggests that, unlike RD2, these strains may regulate IAA biosynthesis through mechanisms independent of bacterial density. It is possible that salinity triggers transcriptional or post-transcriptional changes in genes related to auxin biosynthesis (such as *ipdC*) or that cellular energy is redirected toward stress adaptation rather than metabolite production under saline conditions [51,53,54]. This indicates that the reduction in IAA production by these bacteria could be linked to other factors, including interference in the tryptophan-dependent IAA anabolic pathway, rather than to the number of cells that multiplied (Table 5). In two of the tryptophan-dependent IAA biosynthesis metabolic pathways in bacteria, indole-3-pyruvic acid (IPyA) and indole-3-acetamide (IAM) are used as intermediates. In the IPyA pathway, tryptophan is converted into IPyA by aminotransferases encoded by the *TAM* genes; subsequently, IPyA is converted into indole-3-acetaldehyde (IAAId) by the enzyme pyruvate decarboxylase (PDC), which is encoded by the indole-3-pyruvate decarboxylase (*ipdC*) gene. In the last step, IAAId is converted into IAA by the enzyme aldehyde dehydrogenase (ALDH) [53]. The activity of the PDC enzyme and the expression of the *ipdC* gene are affected by abiotic stress factors, which include aerobic conditions in the growth medium, salinity, carbon source, and the addition of tryptophan as a precursor [51,54,55]. The expression of the *ipdC* gene in bacteria and the concentration of IAA increase when tryptophan is supplied to the growth medium [50]. Under salt stress (NaCl), bacteria can survive and maintain cell density but reduce intracellular IAA production and its export to the external environment as a result of decreased expression of the *ipdC* gene [53]. This effect could be associated with the response of strains RD4, RD17, RD27, and JLB4 to adapt to salinity.

### 3.7. Antioxidant Activity

**Figure 4:**
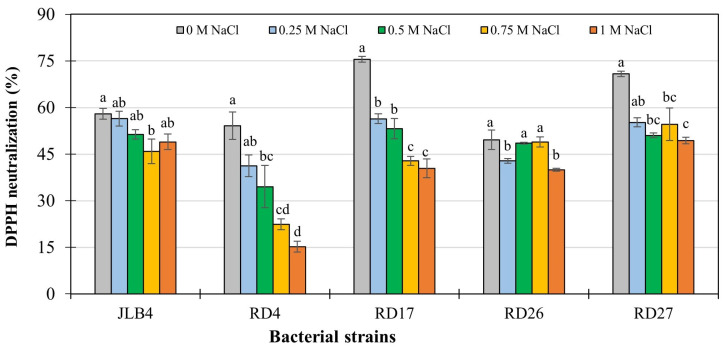
Non-enzymatic antioxidant capacity of filtrates from four bacterial strains isolated from the rhizosphere of saltgrass (*Distichlis spicata*) and the reference strain JLB4 (*Arthrobacter pokkalii*) under normal and salt stress (NaCl) conditions. Means ± SE with different letters above the bar indicate statistically significant differences between treatments (Tukey, *p* ≤ 0.05), n = 6.

Antioxidants are compounds that delay or prevent oxidative damage to certain molecules in the presence of free radicals. These molecules donate electrons to free radicals, rendering them harmless and thus minimizing their interference with the metabolic pathways of organisms [56]. The DPPH neutralization capacity in nutrient broth showed that the antioxidant activity of the bacteria was affected by the addition of NaCl. In strains RD4, RD17, and RD27, the highest antioxidant activity was present under normal growth conditions, and the lowest activity was observed with the addition of 1 M NaCl. Similarly, in strain JLB4, there was no significant reduction in antioxidant activity under salt stress, while in strain RD26, the percentage of DPPH neutralization was affected in the presence of 0.25 and 1 M NaCl (Figure 4). The reduction in antioxidant activity observed under saline conditions suggests that oxidative stress affects the extracellular metabolism of most isolates. However, the maintenance of relatively higher DPPH scavenging capacity in strains RD26 and RD27 indicates a potential adaptive mechanism to salinity. This non-enzymatic antioxidant response may protect cells from damage caused by ROS and preserve bacterial viability and function under stress. Similar mechanisms have been reported in halotolerant bacteria such as *Klebsiella variicola* and *Bacillus mycoides*, which produce antioxidant metabolites to cope with saline-induced oxidative stress and enhance their plant growth-promoting effects [11,12].

While the DPPH assay provided an initial estimate of radical scavenging capacity, this method does not allow the identification of specific antioxidant compounds. Therefore, a deep characterization of individual metabolites involved in this activity, such as phenazines, flavins, or glutathione, and the corresponding evaluation of their ecological stability and functional relevance in the rhizosphere are still lacking and need to be further investigated.

The rhizosphere of halophytes is an important reservoir of bacteria that can tolerate high salt concentrations and show great potential for use in agriculture. To achieve such an application in crops, the first step is to identify the mechanisms that promote in vitro growth under specific environmental conditions. This allows the qualitative and quantitative identification of strains that are of particular interest for decision-making regarding their application [57]. The bacteria obtained in this research and characterized in vitro are highly tolerant to salinity, and some showed a high capacity to produce IAA, siderophores, and EPS. The anabolic pathway of IAA in plants is affected by salinity [58], so the five bacterial strains that showed the greatest capacity for IAA synthesis are excellent candidates for inoculation in crops established under normal conditions or under salinity stress. Not all strains that show stimulatory mechanisms in vitro can improve plant growth [57]. Therefore, the five selected bacterial strains were evaluated on tomato seedlings under greenhouse conditions. The results of this evaluation, along with the molecular identification of the most promising strains, are presented below, highlighting their potential for applications in crops exposed to salinity.

### 3.8. Plant Growth Promotion Induced by Bacteria in Tomato Seedlings Under Greenhouse Conditions

Under greenhouse conditions, bacterial inoculation significantly affected tomato seedling growth. Differences were observed in stem diameter, leaf area, and dry biomass of shoots and roots (Table S1, Supplementary Materials).

Freshly harvested plants contain between 70% and 95% water. However, once this water evaporates, the resulting dry biomass is determined by the presence of carbon (C), hydrogen (H), and oxygen (O). These elements account for up to 92% of the dry biomass weight in plants [59]. Since leaf area determines a plant’s capacity for light interception and CO_2_ assimilation, it is a critical determinant of biomass accumulation and vegetative growth. Although the relationship between leaf area growth and plant biomass may vary depending on carbon partitioning strategies, leaf area remains a reliable morphological predictor of biomass accumulation in vegetative plants [60].

**Figure 5:**
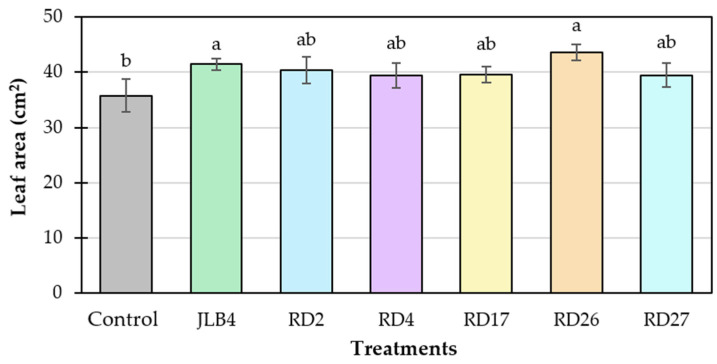
Effect of the inoculation of five bacterial strains isolated from the rhizosphere of saltgrass (*Distichlis spicata*) and the reference strain JLB4 (*Arthrobacter pokkali*) on the leaf area of tomato plants. Means ± SD with different letters above the bars indicate statistically significant differences between treatments (Tukey, *p* ≤ 0.05), *n* = 4.

Inoculation of plants with strains RD2, RD4, RD17, and RD27 did not improve leaf area. In contrast, plants inoculated with strains JLB4 and RD26 exhibited 15.8 and 21.9% greater leaf area, respectively, compared to the uninoculated control plants, suggesting a positive influence of these strains on the development of foliar structures (Figure 5).

Biometric variables such as stem diameter and dry biomass are widely used to assess seedling quality in horticultural crops [61].

Bacterial inoculation had a remarkable influence on the morphological traits of tomato seedlings grown under greenhouse conditions. Among the evaluated parameters, stem diameter was significantly enhanced by several bacterial strains. Strain RD4 exhibited the strongest effect, promoting a stem thickness approximately 9.4% higher than that of the uninoculated control. Other strains, such as RD26 and RD27, also stimulated stem development, with increases of around 6%, while strain RD2 showed only a slight improvement and did not differ significantly from the control (Table 6). Stem diameter is recognized as a relevant morphological trait associated with seedling vigor [62], and in tomato, it has also been linked to water status and overall growth potential [63].

Regarding shoot dry biomass, the most pronounced effect was observed in plants inoculated with strain RD26, which exhibited an increase of approximately 30% compared to uninoculated controls. Similarly, strains RD27 and RD4 promoted significant biomass accumulation, with increases of 25 and 23%, respectively, highlighting their potential as effective plant growth-promoting bacteria. Conversely, plants treated with strain RD2 and the reference strain JLB4 showed moderate improvements, while the lowest shoot biomass was recorded in non-inoculated seedlings (Table 6). The accumulation of dry biomass in higher plants serves as an indirect indicator of the nutrient absorption capacity of the roots and the uptake of CO_2_ by the leaves, along with its fixation into carbon compounds during the dark phase of photosynthesis, which takes place in the stroma of the chloroplasts [64].

Of root dry biomass, only strain RD26 promoted a statistically significant increase, boosting root biomass by over 27% relative to the control. The remaining strains, including JLB4, did not show significant differences, suggesting that RD26 uniquely contributed to root system development under the experimental conditions (Table 6). A well-developed root system provides better anchorage and facilitates the supply of water and nutrients, both in nurseries and in the field, while also offering greater protection against biotic and abiotic stresses. These factors are reflected in improved plant growth, increased dry biomass accumulation, and higher crop yields [65]. Therefore, a larger root volume enhances the plant’s ability to acquire H and O from water, which are the main components of the organic compounds that constitute both the fresh and dry matter of plants [59].

### 3.9. Molecular Identification of Effective Strains

The 16S rRNA gene sequences of the selected strains revealed that RD26 shared 99.69% similarity with *Pseudomonas* sp., while RD27 exhibited 99.86% similarity with *Zhihengliuella halotolerans*. These identifications confirm the taxonomic affiliation of the strains with known plant growth-promoting bacteria. The sequences were submitted to the GenBank database and are available under the accession numbers PP446630.1 for RD26 and PP446631.1 for RD27, respectively.

## 4. Conclusions

In this study, 29 bacterial strains (RD1 to RD29) were isolated from the rhizosphere of *Distichlis spicata* growing in highly saline-sodic soil (pH 10.8, EC 30.34 dS m^−1^, ESP 58.8). Among them, 24 strains demonstrated in vitro salt tolerance, and five (RD2, RD4, RD17, RD26, and RD27) stood out for their ability to synthesize indole-3-acetic acid (IAA), produce siderophores, and exhibit antioxidant activity, even under saline stress.

Although salinity negatively affected IAA production and antioxidant capacity in most strains, several retained significant metabolic activities, suggesting effective stress-adaptive mechanisms. Halotolerant strains may modulate osmotic balance and reduce oxidative stress through the synthesis of compatible solutes, exopolysaccharides (EPS), or reactive oxygen species (ROS)-scavenging metabolites. These physiological adaptations likely underpin their ability to maintain growth and phytohormone production under NaCl stress.

Greenhouse trials confirmed the potential of these strains to enhance tomato seedling vigor. Strains RD26 and RD27 promoted shoot and root biomass, leaf expansion, and stem thickness, highlighting their promise as plant growth-promoting bacteria (PGPB) under both optimal and saline conditions. Molecular identification revealed RD26 as *Pseudomonas sp.* and RD27 as *Zhihengliuella halotolerans*, two genera known for their resilience and versatile PGP traits.

Overall, the mechanisms involved—such as IAA biosynthesis under osmotic stress, siderophore-mediated nutrient acquisition, and EPS-driven ion exclusion—position these strains as strong candidates for the development of bioinoculants aimed at improving crop performance in salt-affected soils.

## Figures and Tables

**Table 1:** Chemical analysis of soil fertility collected in Montecillo, Texcoco, State of Mexico, Mexico (19°27′56.3″ N, 98°55′2.4″ W, at 2240 m elevation).

Ca	K	Mg	Na	CEC	P	Cu	Fe	Mn	Zn	O. M.	Nt
(cmol_(+)_ kg^−1^)					(mg kg^−1^)					(%)	
17.39	20.22	1.15	45.93	86.95	84.96	0.88	6.34	4.04	0.30	2.41	0.12

CEC = cation-exchange capacity, O. M. = organic matter, Nt = total nitrogen.

**Table 2:** Ion analysis in the saturated paste extract collected in Montecillo, Texcoco, State of Mexico, Mexico (19°27′56.3″ N, 98°55′2.4″ W, at 2240 m elevation).

HCO_3_^-^	CO_3_^2−^	Cl^-^	SO_4_^2−^	B	Na^+^	P	K^+^	Ca^2+^	Mg^2+^
(mg L^−1^)									
2334.01	249.00	3515.62	2122.13	39.77	963.80	31.60	154.56	11.07	2.10

**Table 3:** Salinity tolerance of bacterial strains isolated from the rhizosphere of saltgrass (*Distichlis spicata*) in Montecillo, Texcoco, State of Mexico, Mexico (19°27′56.3″ N, 98°55′2.4″ W, at 2240 m elevation).

Strain	NaCl (M)						
	0	0.25	0.50	0.75	1	2	3
**RD1**	**+**	**+**	**+**	**+**	**+**	**−**	−
**RD2**	**+**	**+**	**+**	**+**	**+**	**−**	−
RD3	+	+	+	+	+	−	−
**RD4**	**+**	**+**	**+**	**+**	**+**	**+**	−
RD5	+	+	+	+	−	−	−
RD6	+	+	+	+	+	−	−
**RD7**	**+**	**+**	**+**	**+**	**+**	**+**	−
**RD8**	**+**	**+**	**+**	**+**	**+**	**+**	−
**RD9**	**+**	**+**	**+**	**+**	**+**	**+**	−
**RD10**	**+**	**+**	**+**	**+**	**+**	**+**	−
**RD11**	**+**	**+**	**+**	**+**	**+**	**+**	−
RD12	+	+	+	+	+	−	−
**RD13**	**+**	**+**	**+**	**+**	**+**	**+**	−
**RD14**	**+**	**+**	**+**	**+**	**+**	**+**	−
**RD15**	**+**	**+**	**+**	**+**	**+**	**+**	−
**RD16**	**+**	**+**	**+**	**+**	**+**	**+**	−
**RD17**	**+**	**+**	**+**	**+**	**+**	**+**	−
**RD18**	**+**	**+**	**+**	**+**	**+**	**+**	−
**RD19**	**+**	**+**	**+**	**+**	**+**	**+**	−
**RD20**	**+**	**+**	**+**	**+**	**+**	**+**	−
**RD21**	**+**	**+**	**+**	**+**	**+**	**+**	−
**RD22**	**+**	**+**	**+**	**+**	**+**	**+**	−
**RD23**	**+**	**+**	**+**	**+**	**+**	**+**	−
**RD24**	**+**	**+**	**+**	**+**	**+**	**−**	−
**RD25**	**+**	**+**	**+**	**+**	**+**	**−**	−
**RD26**	**+**	**+**	**+**	**+**	**−**	**−**	−
**RD27**	**+**	**+**	**+**	**+**	**+**	**+**	−
**RD28**	**+**	**+**	**+**	**+**	**+**	**+**	−
**RD29**	**+**	**+**	**+**	**+**	**+**	**+**	−

+ = positive, − = negative. The bacterial strains highlighted in gray and bold correspond to those selected to identify plant growth-promoting traits.

**Table 4:** Plant growth-promoting traits associated with rhizosphere bacteria of saltgrass (*Distichlis spicata*) isolated from soil in Montecillo, Texcoco, State of Mexico, Mexico (19°27′56.3″ N, 98°55′2.4″ W, at 2240 m elevation).

Strain	Growth-Promoting Traits				
	Siderophores	PS	IAA	EPS	
				Sucrose	Glucose
RD1	−	−	−	−	−
RD2	+	−	+	−	−
RD4	+	−	+	−	+
RD7	−	−	−	−	−
RD8	−	−	−	−	−
RD9	−	−	−	−	−
RD10	−	−	−	+	+
RD11	−	−	−	+	−
RD13	−	−	−	−	−
RD14	−	−	−	−	+
RD15	−	−	−	−	−
RD16	−	−	−	−	−
RD17	+	−	+	−	−
RD18	−	−	−	−	−
RD19	−	−	−	−	−
RD20	−	−	−	−	−
RD21	−	−	−	−	−
RD22	−	−	−	−	−
RD23	−	−	−	−	−
RD24	−	−	−	−	−
RD25	−	−	−	−	−
RD26	+	−	+	−	−
RD27	+	−	+	−	−
RD28	−	−	−	+	−
RD29	−	−	−	+	+

IAA = indoleacetic acid; PS = phosphate solubilization; EPS = exopolysaccharides. + = positive, − = negative. Data were analyzed in triplicate.

**Table 5:** Correlation analysis between indoleacetic acid (IAA) production and bacterial growth of five bacterial strains isolated from the rhizosphere of saltgrass (*Distichlis spicata*) and the reference strain JLB4 (*Arthrobacter pokkalii*) in LB medium under salt stress.

Bacterial Strains	*p* Value Correlation	Correlation Coefficient
JLB4	0.140 ^p^	NS
RD2	<0.001 ^s^	1 ***
RD4	0.350 ^s^	NS
RD17	0.110 ^p^	NS
RD26	0.170 ^p^	NS
RD27	0.100 ^p^	NS

*p*-value correlation of ^s^ = Spearman’s for non-normally distributed data and ^p^ = Pearson’s for normally distributed data. *** = high correlation; NS: not significant.

**Table 6:** Influence of bacterial inoculation on dry biomass weights in tomato plants inoculated with five bacterial strains isolated from saltgrass (*Distichlis spicata*) and a reference bacterial strain (JLB4), 36 days after inoculation.

Treatments	Stem Diameter (mm)	Shoot Dry Biomass (mg)	Root Dry Biomass (mg)
Control	2.24 ± 0.07 c	186.96 ± 18.08 b	64.04 ± 6.60 b
JLB4	2.39 ± 0.02 ab	227.47 ± 6.02 ab	75.26 ± 5.62 ab
RD2	2.31 ± 0.08 bc	221.86 ± 28.39 ab	69.47 ± 8.77 ab
RD4	2.45 ± 0.05 a	229.70 ± 23.13 a	71.46 ± 10.18 ab
RD17	2.36 ± 0.03 abc	224.53 ± 9.86 ab	72.16 ± 4.63 ab
RD26	2.38 ± 0.06 ab	243.15 ± 8.12 a	81.44 ± 5.47 a
RD27	2.38 ± 0.04 ab	233.70 ± 17.76 a	64.92 ± 4.78 b

Means ± SD followed by different letters within the same column indicate significant differences (Tukey, *p* ≤ 0.05), n = 4.

## Data Availability

The original contributions presented in this study are included in the article/Supplementary Materials. Further inquiries can be directed to the corresponding author.

## Supplementary Materials

The following supporting information can be downloaded at https://www.mdpi.com/article/10.3390/microorganisms13092046/s1: Figure S1. Siderophore production of 24 bacterial strains isolated from the rhizosphere of saltgrass (*Distichlis spicata*) and grown in CAS medium, after 5 days of cultivation. Figure S2. Phosphate solubilization assay at 14 d by 24 bacterial strains isolated from the rhizosphere of saltgrass (*Distichlis spicata*) and the reference strain JLB4 (*Arthrobacter pokkalii*). Figure S3. Production of exopolysaccharides (EPS) by 24 bacterial strains isolated from the rhizosphere of saltgrass (*Distichlis spicata*) in LB culture medium supplemented with 5% sucrose. Figure S4. Production of exopolysaccharides (EPS) by 24 bacterial strains isolated from the rhizosphere of saltgrass (*Distichlis spicata*) in LB culture medium supplemented with 5% glucose. Table S1. Analysis of variance (ANOVA) for growth parameters in tomato seedlings inoculated with bacterial strains isolated from the rhizosphere of saltgrass (*Distichlis spicata*).
