# Supplementary material for: Rhizosphere-Associated Bacteria of Saltgrass [Distichlis spicata (L.) Greene] Show Enhanced Ability to Tolerate Saline Environments and Stimulate Plant Growth
Source: Microorganisms. 2025 Sep 2;13(9):2046. doi: 10.3390/microorganisms13092046 (PMC12472804; doi:10.3390/microorganisms13092046)
Supplement: Supplementary file 1 [file microorganisms-13-02046-s001.zip › microorganisms-3772762_Figure_S4.pdf]

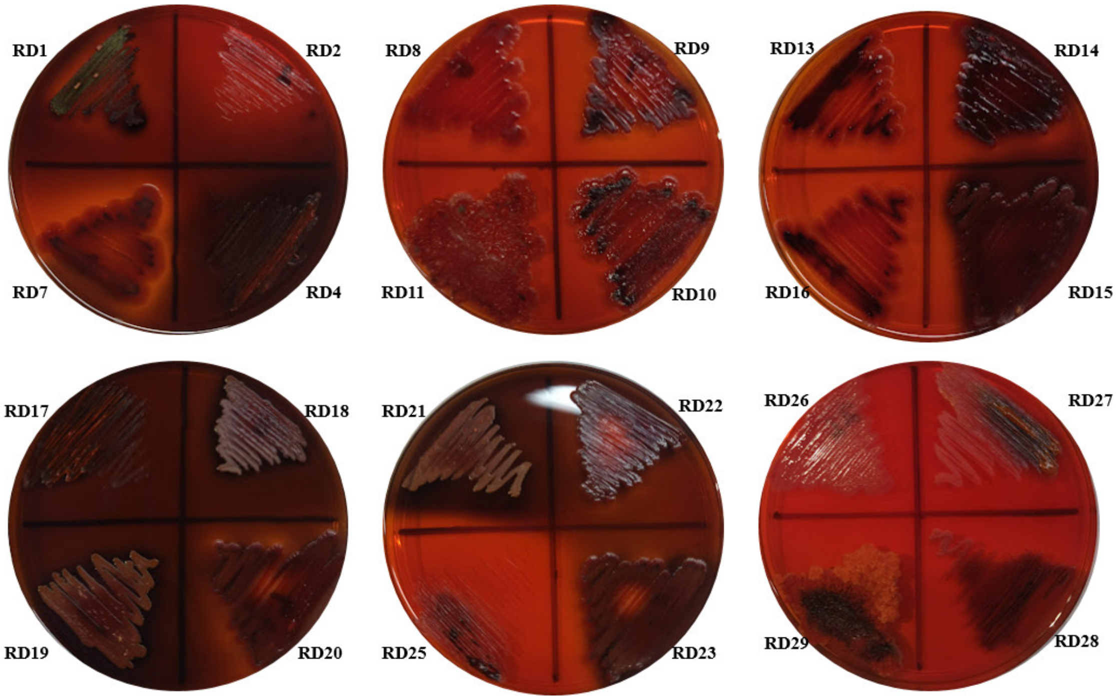

Figure S4. Production of exopolysaccharides (EPS) by 24 bacterial strains isolated from the rhizosphere of saltgrass (*Distichlis spicata*) in LB culture medium supplemented with 5% glucose.
