# Supplementary material for: Rhizosphere-Associated Bacteria of Saltgrass [Distichlis spicata (L.) Greene] Show Enhanced Ability to Tolerate Saline Environments and Stimulate Plant Growth
Source: Microorganisms. 2025 Sep 2;13(9):2046. doi: 10.3390/microorganisms13092046 (PMC12472804; doi:10.3390/microorganisms13092046)
Supplement: Supplementary file 1 [file microorganisms-13-02046-s001.zip › microorganisms-3772762_Table_S1.pdf]

Table S1. Analysis of variance (ANOVA) for growth parameters in tomato seedlings inoculated with bacterial strains isolated from the rhizosphere of saltgrass (*Distichlis spicata*).

Agronomic variable	Degrees of Freedom	Mean Square	Pr > F	Coeffiecient of variation (%)
Stem diameter	6	0.0175	0.0025 *	2.50
Leaf area	6	22.6264	0.0019 *	5.19
Shoot dry weight	6	1254.6398	0.0077 *	7.88
Root dry weight	6	144	0.0310 *	9.90

* Indicates significant differences according to Tukey's test ($p \leq 0.05$).
